# Optimization of TiO_2_ and PMAPTAC Concentrations of a Chemical Humidity Sensing Mechanism

**DOI:** 10.3390/s91007837

**Published:** 2009-09-30

**Authors:** Souhil Kouda, Zohir Dibi, Abdelghani Dendouga, Fayçal Meddour, Samir Barra

**Affiliations:** Laboratoire d'Electronique Avancée, Département d'Electronique, Université de Batna, 05 avenue Chahid Boukhlouf, 05000 Batna, Algeria; E-Mails: zohirdibi@yahoo.fr (Z.D.); dendouga_gh@hotmail.com (A.D.); faycal.meddour@yahoo.fr (F.M.); barrasamir@hotmail.com (S.B.)

**Keywords:** resistive humidity sensor, sensing mechanism, TiO_2_, PMAPTAC, neuronal network, MLP

## Abstract

This work aims to achieve an optimization of the TiO_2_ and PMAPTAC concentrations in a chemical resistive-type humidity sensing mechanism (RHSM). Our idea is based primarily on the modeling of the sensing mechanism. This model takes into account the parameters of non-linearity, hysteresis, temperature, frequency, substrate type. Furthermore, we investigated the TiO_2_ and PMAPTAC effects concentrations on the humidity sensing properties in our model. Secondly, we used the Matlab environment to create a database for an ideal model for the sensing mechanism, where the response of this ideal model is linear for any value of the above parameters. We have done the training to create an analytical model for the sensing mechanism (SM) and the ideal model (IM). After that, the SM and IM models are established on PSPICE simulator, where the output of the first is identical to the output of the RHSM used and the output of the last is the ideal response. Finally a “DIF bloc” was realized to make the difference between the SM output and the IM output, where this difference represents the linearity error, we take the minimum error, to identify the optimal TiO_2_ and PMAPTAC concentrations. However, a compromise between concentrations, humidity and temperature must be performed. The simulation results show that in low humidity and at temperature more than 25 °C, sample 1 is the best (in alumina substrate). However, the sample 9 represents the best sensor (in PET substrate) predominately for the lowest humidity and temperature.

## 1. Introduction

Humidity measurement is one of the important tasks in many industrial product manufacturing processes such as for textiles, foods, paper, semiconductors and petrochemicals. The humidity is a significant parameter like the pressure or the temperature. It changes the electric characteristics of materials and acts on the responses of the performed systems [1,2].

Recently, artificial neural networks (ANNs) have emerged as a highly effective learning technique suitable for performing nonlinear, complex, and dynamic tasks with a high degree of accuracy [3]. Complex nonlinear and cross sensitivity modeling has been successfully tackled with ANNs [4]. Neural models are, therefore, much faster than physics/electro-mechanical models and have a higher accuracy than analytical and empirical models. Furthermore, they are easy to develop for a new device or technology [5,6].

Thus, ANNs are commonly used for smart sensor applications, where the aim is to decrease the sensor errors [7–10]. This work proposes ANNs models for a resistive humidity sensing mechanism (SM) and its ideal model (IM), operating under a dynamic environment. They provide accurate readout of the applied humidity; we have designed and established on PSPICE software the sensing mechanism SM and its ideal model IM. The SM model carried out take into account the non linearity response, the hysteresis, the temperature and frequency effects in a dynamic environment and TiO_2_ and PMAPTAC concentrations effects, the IM model response is linear for any substrate, hysteresis values, temperature values, frequency values and concentrations. The linearity error is the difference between the SM output and the IM output, where, the optimal concentrations of the TiO_2_ and the PMAPTAC are taking at the minimum nonlinearity error; these optimal concentrations give the best sensor.

### 2. Resistive Humidity Sensor Mechanism (RHSM) Design

The sensing mechanism measures the change in electrical impedance, it absorbs the water vapor then ionic functional groups are dissociated, resulting in an increase in electrical conductivity. The impedance range of typical resistive elements varies from 100 to 100,000,000 ohms.

The resistive-type humidity sensing mechanism used for this modeling was fabricated by the *in situ* photopolymerization of TiO_2_ nanoparticles/polypyrrole (TiO_2_ NPs/PPy) and TiO_2_ nanoparticles/ polypyrrole/poly-[3-(methacrylamino)propylrsqb; trimethylammonium chloride (TiO_2_ NPs/PPy/PMAPTAC) composite thin films, our model contain tow mechanisms of this composite, the first is fabricated on a polyester (PET) substrate and the second on an alumina substrate. The effect of the TiO_2_ and PMAPTAC concentrations on the humidity sensing properties are investigated.

The various compositions are shown in Table 1. The composite solution is coated, on an alumina and onto a PET substrate, with a pair of comb-like electrodes.

## 3. Analytical Model SM

The experimental results were used [11,12] to create a database arranged as (Sub, Hys, T, F, TiO_2_, PMA, H, Z), where Sub is the substrate sensing mechanism type, Hys is the hysteresis, T is the environment temperature in the measurement point, F is the applied frequency, TiO_2_ is the TiO_2_ concentration, PMA is the PMAPTAC concentration, H is the humidity applied to the SM, and Z is the SM response. Note here that, in our model, the input Sub takes the value of 0 for the alumina substrate and the value 1 for the PET substrate and the input Hys takes the value 0 for humidification and the value 1 for desiccation. In a second step we arrange the data into training, validation, and test subsets. One-fourth of the data are taken for the validation set, one-fourth for the test set, and half for the training set. The sets are picked as equally spaced points throughout the original data. It is important not to use any element of the test base and validation base throughout all training. These bases are reserved only for the final performance measurement [13].

### 3.1. Training

**Figure 1.**
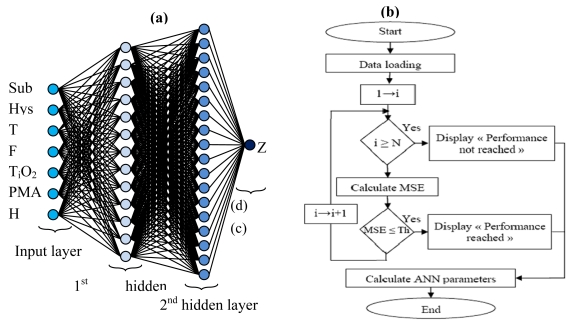
**(a)** Symbolic notation of the ANN optimized model **(b)** Training program flowchart.

The training phase requires a database (eight vectors “seven inputs and one output”, 2,700 elements by vector for the training base and 1,296 elements by vector for the validation and test base), selecting the network architecture and finding the numbers of layers and neurons in each layer. However, since the neuron numbers in the input and output layers are determined by the input and output numbers of the system to be modeled, the SM has 8 inputs and only one output Z “Resistance”, the input layer has 8 neurons and only one neuron for the output layer. So that the ANN model accurately expresses the SM response variation, it is a question of finding the optimal number of the hidden layers, the number of neurons by layer and the transfer function. After many tests of different ANN models we considered MLP with two hidden layers, 13 neurons and the transfer function Logsig for the first layer, 17 neurons and the Transfer function Logsig for the second layer and the Transfer function Linear for the output layer. Figure 1(a) shows the symbolic notation of ANN optimized model and Table 1 summarized those parameters.

We have made the neuronal network training for the database with the back propagation (BP) algorithm; Figure 1(b) shows the program flowchart.

Note that the data loading is: training base, test base, number of layers and neurons, type of the transfer functions, number of iteration and estimate threshold. N is the number of iterations, MSE is the mean square error, Th is the estimate threshold “Test MSE” and the ANN parameters are the neuronal network element (Bni the bias matrix and Wnji the weights matrix). Finally we measure the model performance obtained with the test base.

### 3.2. Model Test

**Figure 2.**
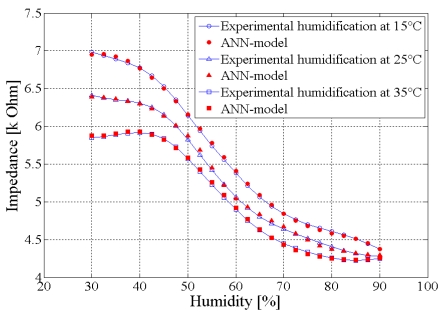
ANN model performance for the sample 1, measured at 1 KHz at fixed temperatures 15, 25 and 35 °C.

The comparison between the initial database and that obtained after the training, using the test base, indicates that our model expresses accurately the response variation of the RHSM. Figure 2 presents the model performance obtained for the sample 1, measured at 1 KHz at fixed temperatures 15, 25 and 35 °C.

## 4. Implementation ANN in PSPICE

The SM was modeled using the ABM (Analog Behavioral Modeling) of the PSPICE library.

## 5. Ideal Model

The IM of the SM has the same inputs of the SM however its output is linear, we realize a program using Matlab environment to obtain the database of this output which is expressed by the equation:

(1) $$\text{Z}(\text{Sub},\text{Hys},\text{ T},\text{ F},\text{ Ti}{\text{O}}_{2},\text{PMA})=\text{AH}+\text{B}$$

Where Z is the impedance value (KΩ) for the substrate (Sub) type and hysteresis (Hys) state, at the temperature (T) (°C) and frequency (F) (KHz), with TiO_2_ and PMAPTAC (PMA) concentrations (g), H is the humidity (%), A and B are the linear equation constants determinate by Max Z, Min Z and the range of humidity variation.

The generation of training base, test base and validation base is similar to that of the SM model. However, in the IM model linearized humidity is taken as the desired output. In the simulation study, the same MLP with 13-17-1 structure of the SM was chosen for the IM. This later was trained in a similar manner as in the case of SM model.

## 6. Simulation Results

### 6.1. SM Validation

**Figure 4.**
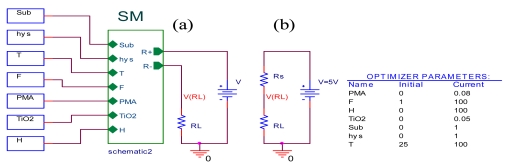
**(a)** The humidity sensor electrical circuit **(b)** The measurement circuit of the sensor resistance.

In order to validate the sensor introduced on PSPICE simulator, the SM is implemented in the electrical circuit as shown in Figure 4(a) and the measurement circuit of sensor resistance is shown in Figure 4(b). The sensor resistance Rs may be calculated with the equation:

(2) $$R_{S}=\left(\frac{V-V_{\text{RL}}}{V_{\text{RL}}}\right)\times R_{L}=\frac{\left(V-V_{\text{RL}}\right)}{I_{\text{RL}}}$$

**Figure 5.**
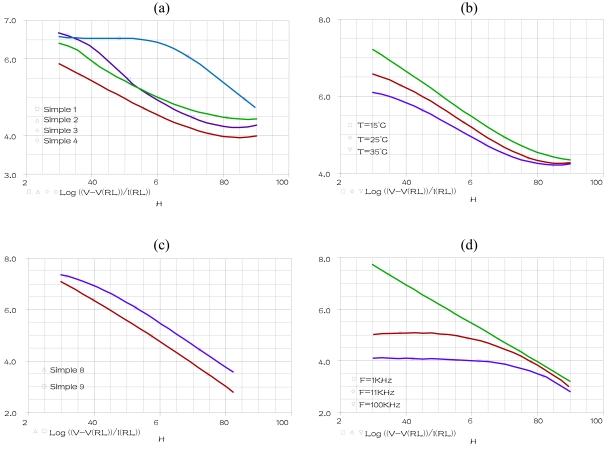
**(a)** SM output for a parametric SWEEP analysis for sample 1–4 at fixed temperature and frequency 25 °C, 1 KHz respectively **(b)** SM output for a parametric SWEEP analysis for the sample 1 at fixed frequency 1KHz, for the temperatures 15, 25 and 35 °C **(c)** SM output for a parametric SWEEP analysis for sample 8–9 at fixed temperature and frequency 25 °C, 1 KHz respectively **(d)** SM output for a parametric SWEEP analysis for the sample 1 at fixed temperature 25 °C, for the frequencies 1, 10 and 100 KHz.

The temperature, the frequency and the PMA are fixed at 25 °C, 1 KHz and 0 (g) respectively when humidity is varying within the range 30% to 90%, for humidification (Hys = 0) in the alumina substrate (Sub = 0). A parametric SWEEP analysis, for the four concentrations values of TiO_2_ 0, 0.0012, 0.0118 and 0.048 (g) (concentrations values of samples 1–4) gives the results represented Figure 5(a).

A parametric SWEEP analysis, for the three temperatures 15, 25 and 35 °C, at fixed frequency of 1 KHz when humidity is varying within the range 30% to 90%, for the sample 1 in humidification, gives the results represented in Figure 5(b).

The temperature, the frequency and the TiO_2_ are fixed at 25 °C, 1 KHz and 0.048 (g) respectively when humidity is varying within the range 30% to 90%, for (Hys = 1) in the PET substrate (Sub = 1). A parametric SWEEP analysis, for the tow PMA concentrations values of 0.008 and 0.08 (g) (concentrations values of samples 8–9) gives the results represented Figure 5(c).

A parametric SWEEP analysis, for the three frequencies 1, 11 and 100 KHz, at fixed temperatures of 25 °C when humidity is varying within the range 30% to 90%, for the sample 9 in humidification, gives the results represented in Figure 5(d).

These simulations indicate that our component, introduced in PSPICE simulator, expresses accurately the response variation of the SM compared to the [11,12] experimental results.

### 6.2. IM Validation

**Figure 6.**
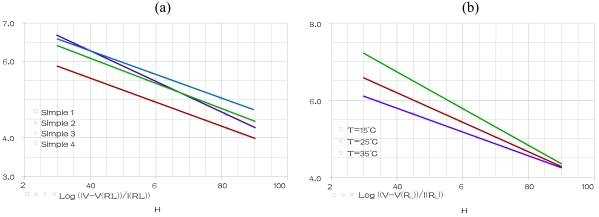
**(a)** SM output for a parametric SWEEP analysis for samples 1–4 at fixed temperature and frequency 25 °C and 1 KHz respectively **(b)** SM output for a parametric SWEEP analysis for the sample 1 at fixed frequency 1KHz, for the temperatures 15, 25 and 35 °C.

By analogy to the first test, we carried out a second test for the ideal model. The temperature, the frequency and the PMA are fixed at 25 °C, 1 KHz and 0 (g) respectively when humidity is varying within the range 30% to 90%, for humidification (Hys = 0) in the alumina substrate (Sub = 0). A parametric SWEEP analysis, for the samples 1–4 gives the results represented Figure 6(a).

A parametric SWEEP analysis, for the three temperatures 15, 25 and 35 °C, at fixed frequency of 1 KHz when humidity is varying within the range 30% to 90%, for the sample 1 in humidification, gives the results represented in Figure 6(b).

These simulations indicate that our component, introduced in PSPICE simulator, expresses accurately the ideal response “a linear response for any substrate, hysteresis values, temperature values, frequency values and TiO_2_ and PMA concentrations”.

### 6.3. DIF Bloc Validation

**Figure 7.**
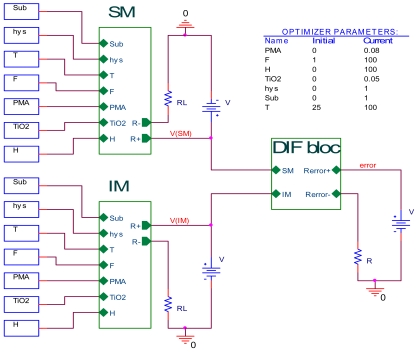
Electrical circuit.

In order to validate our work, introduced on PSPICE simulator, the sensing mechanism model SM and its ideal model IM with the DIF bloc have been implemented in the electrical circuit shown as in Figure 7.

The “DIF bloc” is realized to make the difference between SM output and IM output, where this difference is expressed by the equation:

(3) $$R_{\text{error}}=R_{\text{IM}}-R_{\text{SM}}$$

We can rewrite Equation (3) as:

(4) $$R_{\text{error}}=\left[\left(\frac{V-V_{\text{SM}}}{V_{\text{SM}}}\right)-\left(\frac{V-V_{\text{IM}}}{V_{\text{IM}}}\right)\right]\times R_{L}$$

**Figure 8.**
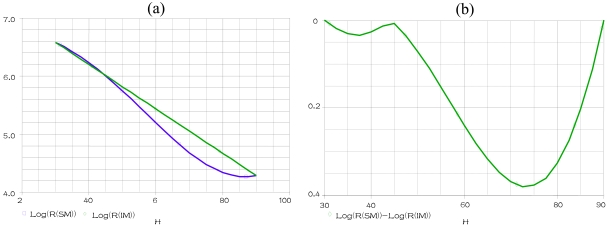
**(a)** The delivered resistances R_SM_ and R_IM_, by a DC SWEEP analysis for sample 1 **(b)** The delivered resistance Rerror ‖ (Log(R(SM)-Log(IM))) ‖, by a DC SWEEP analysis for sample 1.

The TiO_2_ and PMA concentrations are fixed at 0(g) in the alumina substrate (sample 1), for the temperature 25 °C, at fixed frequency of 1 KHz when humidity is varying within the range 30% to 90%, for the humidification, gives the results represented in Figure 8(a). This simulation shows the SM response and the IM linear response for sample 1, when humidity is varying within the range 30% to 90%. The Figure 8(b) shows the nonlinearity abslute values of the sample 1, at fixed temperature of 25 °C and frequency of 1 KHz, when humidity is varying within the range 30% to 90%.

**Figure 9.**
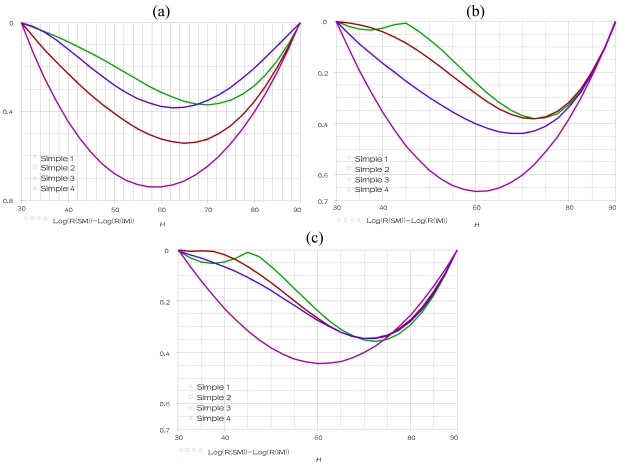
Parametric SWEEP analysis for samples 1–4, at fixed temperature **(a)** 15 °C. **(b)** 25 °C. **(c)** 35 °C.

This simulation shows the sensor's nonlinearity for sample 1, at fixed temperature of 25 °C and frequency of 1 KHz, when humidity is varying within the range 30% to 90%. The temperature is fixed at 15 °C, 25 °C and 35 °C respectively, a parametric SWEEP analysis, for the samples 1–4 when humidity is varying within the range 30% to 90%, gives the results represented in Figure 9.

**Figure 10.**
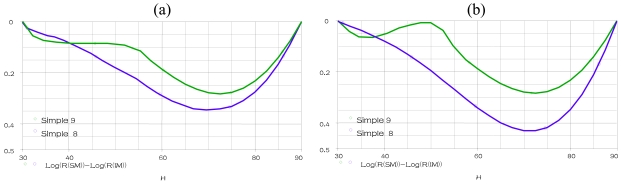
Parametric SWEEP analysis for samples 8–9, at fixed temperature **(a)** 15 °C **(b)** 35 °C.

When the temperature is fixed at 15 °C and 35 °C respectively, a parametric SWEEP analysis, for the samples 8–9 when humidity is varying within the range 30% to 90%, gives the results represented in Figure 10.

These simulations show that the realized circuit is able to show us the nonlinearity (Rerror) response of the humidity sensing mechanism SM for any temperature values, frequency values and TiO_2_ or PMAPTAC concentrations. We compare in these figures the nonlinearity of the four samples 1–4 (samples in alumina substrate) at different temperatures (15 °C, 25 °C and 35 °C). The minimum nonlinearity is reached at the 0(g) of TiO_2_ (sample 1), where this sensor gives the lowest nonlinearity for the humidity range 30% to 45% at temperature more than 25 °C. However, the sample 9 represents the best sensor (in PET substrate) predominately for the lowest humidity and temperature.

## 7. Conclusions

In this paper, we have proposed an electrical circuit used to optimize the TiO_2_ and PMAPTAC concentrations of a resistive humidity sensing mechanism. When the ambient temperature changes over a wide range, the nonlinear response characteristics of the SM undergo change in a complex manner. At different concentrations of TiO_2_ and PMAPTAC, a data points from the sensor characteristics were obtained. Those data were then used to train the MLP model using the back propagation algorithm. After training, the MLP, our model is able to estimate the sensor's response for each substrate and hysteresis type, different TiO_2_ and PMAPTAC concentrations, temperature values and frequency values. It accurately expresses the SM nonlinear characteristics and its dependence on temperature. We use the Matlab environment to create a database for the ideal model; however, its output is linear. This later was trained in a similar manner as in the case of SM model. After that we use the bias matrix and the weights matrix obtained by training to establish our models on PSPICE simulator, which verified the sensors responses, by simulations results. A ‘DIF bloc’ is realized to make the difference between the SM output and the IM output, which represents the nonlinearity. It appears clearly in this study that a sensing mechanism depends on the TiO_2_ and PMAPTAC concentrations. The good response for the alumina substrate (low nonlinearity) is obtained at a small TiO_2_ concentration, for the PET substrate the good response is obtained for the big PMAPTAC concentration. However, the mechanism shows a light temperature dependence at these values, a compromise between concentration and temperature must be performed, we can do the same simulation for different frequencies by this circuit. We can also extend this idea to optimize other parameters, for example, the frequency for Na- and K-montmorillonite based humidity sensors or possibly for different sensing mechanisms types like strip light for MEMS humidity or pressure sensing mechanisms and concentration of Ppy film for low humidity QCM sensor “quartz crystal microbalance”.

## Figures and Tables

**Figure 3.**
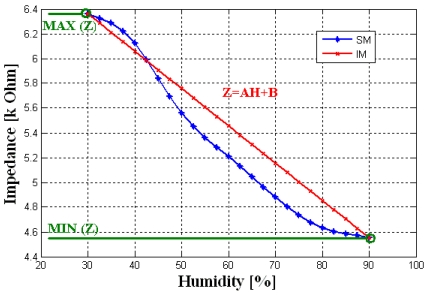
Ideal model output for sample 3 at T = 25 °C and F = 1 KHz in the humidification state.

**Table 1.** Composition of the composite films used to prepare humidity sensors (data taken from [11,12]).

**Substrate**	**Sample number**	**Pyrrole (g)**	**AgNO3 (g)**	**TiO2 (g)**	**PMAPTAC (g)**
alumina	1	0.125	0.0314	0	0
	2	0.125	0.0314	0.0012	0
	3	0.125	0.0314	0.0118	0
	4	0.125	0.0314	0.0480	0
PET	5	0.125	0.0314	0.0012	0
	6	0.125	0.0314	0.0118	0
	7	0.125	0.0314	0.048	0
	8	0.125	0.0314	0.048	0.008
	9	0.125	0.0314	0.048	0.08

**Table 2.** Optimized parameters of the neural networks model.

Database		Training base				21,600
		Test base				10,368
		Validation base				10,368

Number of Neurons		Input layer				8
		1st hidden layer				13
		2nd hidden layer				17
		Output layer				1

Transfer function		1st hidden layer				Logsig
		2nd hidden layer				Logsig
		Output layer				Linear

Input	In (unit)	T(°C)	F(KHz)	TiO2(g)	PMA(g)	H(%)

	Max	35	100	0.048	0.08	90
	Min	15	1	0	0	30

Output		log(Z/Ω)					

		Max			7.7598		
		Min			1.9983		

Test MSE		10-4 (chosen by the user)				

Training MSE		9.589 10-4 (given by the Matlab)				
